# Coordination of Necessary and Permissive Signals by PTEN Inhibition for CNS Axon Regeneration

**DOI:** 10.3389/fnins.2018.00558

**Published:** 2018-08-13

**Authors:** Jie Zhang, Dakai Yang, Haoliang Huang, Yang Sun, Yang Hu

**Affiliations:** ^1^Department of Ophthalmology, Stanford University School of Medicine, Palo Alto, CA, United States; ^2^Department of Ophthalmology, Union Hospital, Tongji Medical College, Huazhong University of Science and Technology, Wuhan, China

**Keywords:** axon regeneration, PTEN/PI3K/Akt, optic nerve, mTOR, GSK3β

## Abstract

In the nearly 10 years since PTEN was identified as a prominent intrinsic inhibitor of CNS axon regeneration, the PTEN negatively regulated PI3K-AKT-mTOR pathway has been intensively explored in diverse models of axon injury and diseases and its mechanism for axon regeneration is becoming clearer. It is therefore timely to summarize current knowledge and discuss future directions of translational regenerative research for neural injury and neurodegenerative diseases. Using mouse optic nerve crush as an *in vivo* retinal ganglion cell axon injury model, we have conducted an extensive molecular dissection of the PI3K-AKT pathway to illuminate the cross-regulating mechanisms in axon regeneration. AKT is the nodal point that coordinates both positive and negative signals to regulate adult CNS axon regeneration through two parallel pathways, activating mTORC1 and inhibiting GSK3ββ. Activation of mTORC1 or its effector S6K1 alone can only slightly promote axon regeneration, whereas blocking mTORC1 significantly prevent axon regeneration, suggesting the necessary role of mTORC1 in axon regeneration. However, mTORC1/S6K1-mediated feedback inhibition prevents potent AKT activation, which suggests a key permissive signal from an unidentified AKT-independent pathway is required for stimulating the neuron-intrinsic growth machinery. Future studies into this complex neuron-intrinsic balancing mechanism involving necessary and permissive signals for axon regeneration is likely to lead eventually to safe and effective regenerative strategies for CNS repair.

## Introduction

**FIGURE 1:**
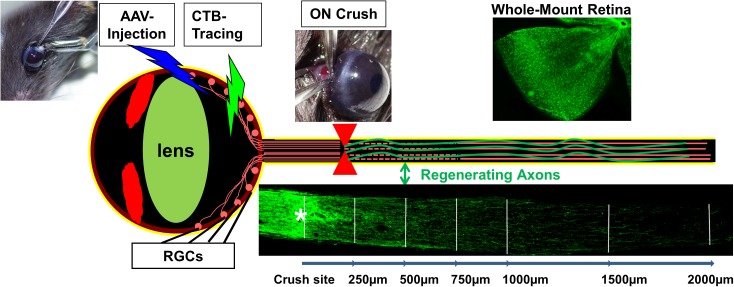
RGC/ON crush provides a straightforward *in vivo* CNS axon injury/regeneration model with clear readout, easy access and amenable to genetic manipulation. Mouse eye can be injected with AAV for genetic manipulation of RGCs and fluorescence-labeled CTB for regenerating axon tracing. Whole-mount retina is for RGC detection and ON longitudinal cryostat section is for visualizing regenerating axons.

Axon injury is a frequent consequence of trauma and a common early feature of CNS degenerative diseases causing life-long neurological deficits. Injuries of CNS axons often result in loss of vital functions because CNS axons fail to regenerate in adult mammals (Schwab and Bartholdi, 1996; Goldberg et al., 2002b; Fitch and Silver, 2008). Both the diminished intrinsic regenerative capacity of mature neurons (Park et al., 2010) and the inhibitory environment of the adult CNS (Yiu and He, 2006) contribute to the growth failure. Neutralizing extracellular inhibitory molecules genetically or pharmacologically yields only limited regeneration and functional recovery (Lee et al., 2010), highlighting the critical importance of neuron-intrinsic factors (Benowitz et al., 2017). To explore the intrinsic regenerative signaling molecules, mouse retinal ganglion cell (RGC) and optic nerve (ON) provide a valuable *in vivo* neural injury system that is relatively simple but robustly replicates CNS traumatic injury and permits straightforward interpretation (**Figure 1**).

Retinal ganglion cells are the only projection neurons in retina to relay visual information from retina to brain. The ON is formed by the projection axons sent exclusively from RGCs; it has the simplicity of an unidirectional axon pathway, which ensures that any nerve fibers passing through the complete crush site are regenerated and do not represent spared axons that underwent collateral sprouting. Adeno-associated viruses (AAV) can be injected directly into the vitreous chamber of the eye to express transgenes specifically and efficiently in adult RGCs. This spatially and temporally controlled genetic manipulation allows us to overcome developmental issues associated with germ line manipulation and to test interventions that can potentially be translated to therapies (Hu, 2015). Exploiting the anatomical and technical advantages of the RGC/ON crush model, multiple signal transduction pathways and transcriptional factors have been linked with CNS axon regeneration (Benowitz et al., 2017; Mahar and Cavalli, 2018). Here we focused on the PTEN/mTOR pathway that we and others have conducted an extensive molecular dissection of the cross-regulating mechanisms in axon regeneration that involve the downstream effectors of PTEN and PI3K to understand the intrinsic mechanisms of CNS axon regeneration (Park et al., 2008; Yang et al., 2014; Guo et al., 2016; Miao et al., 2016; Al-Ali et al., 2017).

## PTEN Deletion Promotes Significant CNS Axon Regeneration

**FIGURE 2:**
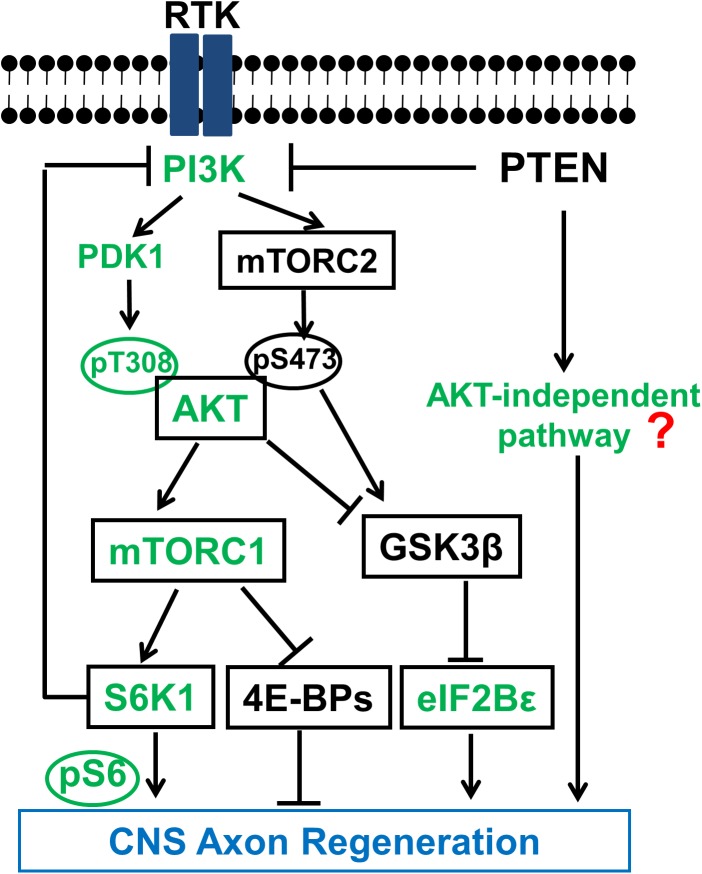
Schematic illustration of the PTEN regulated PI3K-AKT-mTOR signaling pathways in CNS axon regeneration. AKT is positively regulated by the PI3K/PDK1 pathway through phosphorylation of T308, and negatively regulated by the PI3K/mTORC2 pathway through phosphorylation of S473, through at least partially regulation of GSK3β phosphorylation and inhibition. Both AKT downstream effectors, activation of mTORC1 and phosphorylation/inhibition of GSK3β, synergistically promote axon regeneration; inhibition of GSK3β alone is also sufficient for axon regeneration, at least partially through eIF2B𝜀. The activation of mTORC1 and its substrates 4E-BP and S6K is necessary but not sufficient for potent axon regeneration. The question mark represents unknown effectors downstream of PTEN that are AKT-independent and sufficient to initiate CNS axon regeneration, and which potentially interact with the translational targets of mTORC1 to promote potent axon regeneration. Green color-coated molecules are pro-axon regeneration and dark color-coated molecules are anti-axon regeneration.

The initial efforts to understand the intrinsic mechanisms of regenerative failure have led us to postulate that CNS neurons tightly regulate the evolutionarily conserved molecular pathways that control cell growth (Weinberg, 2007) to prevent overgrowth when development is complete. We subsequently used the mouse ON crush model to screen multiple tumor suppressor genes and discovered that deletion of phosphatase and tensin homolog (PTEN), but not Rb (retinoblastoma), P53, Smad4, or LKB1 (liver kinase B1), promotes significant ON regeneration and RGC survival (Park et al., 2008). PTEN, a lipid phosphatase, is a major negative regulator of the phosphatidylinositol 3-kinase (PI3K)-mammalian target of rapamycin complex 1 (mTORC1) pathway (**Figure 2**). Similar axon regeneration phenotypes after PTEN deletion have been reported for mouse cortical motor neurons (Liu et al., 2010; Jin et al., 2015), drosophila sensory neurons (Song Y. et al., 2012) and *Caenorhabditis elegans* motor neurons (Byrne et al., 2014), presumably through activating PI3K-mTORC1-controlled cell growth. Direct activation of mTORC1 also promotes axon regeneration in dopaminergic neurons (Kim S.R. et al., 2011) and RGCs (Duan et al., 2015; Bei et al., 2016; Lim et al., 2016) and peripheral nerves (Abe et al., 2010), providing further support for the critical role of PI3K-mTORC1 in axon regeneration. However, deregulated PTEN/mTOR activities have been implicated in various disorders including metabolic diseases, tumor formation and even senescence (Zoncu et al., 2011). Presumably, uncontrolled or non-specific activation of mTOR and protein synthesis may result in severe negative consequences, such as tumor formation or cognitive impairment. However, not all tumor suppressor genes involved in axon regeneration in our initial screen (Park et al., 2008), indicating the unique role of PTEN/mTOR in determining the neuronal intrinsic regenerative ability. It is scientifically intriguing and clinically important to carry out a molecular dissection of the PTEN/mTOR pathway to acquire unambiguous understanding of the roles of their downstream signaling molecules in axon regeneration, in hope of differentiating to their tumorigenesis roles.

## The Necessary Role of mTORC1 in CNS Axon Regeneration

PI3K is a lipid kinase which can be activated by growth factors, such as insulin and insulin-like growth factor-1 (IGF1), through receptor tyrosine kinase (RTK) (**Figure 2**). PI3K phosphorylates phosphatidylinositol 4,5-bisphosphate (PIP_2_) to produce phosphatidylinositol (3,4,5)-triphosphate (PIP_3_) in the lipid membrane. PIP_3_ in turn recruits AKT to the membrane to be phosphorylated at T308 and activated by phosphoinositide-dependent kinase-1 (PDK1) (Manning and Cantley, 2007). One of the multiple AKT downstream effectors is the complex formed by tuberous sclerosis 1 and 2 (TSC1/TSC2) heterodimer, the negative regulator of mTORC1. AKT activation removes the inhibition of TSC and activates mTORC1. PTEN converts PIP_3_ to PIP2 and thus inhibits the activation of AKT and its downstream effectors. PTEN deletion therefore results in constitutive activation of the PI3K-AKT-mTORC1 pathway, suggesting an important role of mTORC1 in the intrinsic regenerative ability of injured adult CNS neurons. Consistently, rapamycin, an inhibitor of mTORC1, blocked PTEN knockout (KO)-induced ON regeneration (Park et al., 2008), indicating mTORC1 activation is required for axon regeneration.

The functional complex mTORC1 regulates cell growth, proliferation, metabolism, motility and survival (Ma and Blenis, 2009; Laplante and Sabatini, 2012) and its downstream effectors are potential targets for promoting axon regeneration and functional recovery after CNS injury. Unfortunately, the clinical usefulness of mTORC1 activation is limited by the threat of deleterious side effects such as malignancy and cognitive deficits due to uncontrolled protein synthesis and cell proliferation (Laplante and Sabatini, 2012; Song M.S. et al., 2012). We studied the two best-characterized downstream signaling molecules of mTORC1, ribosomal protein S6 kinase (S6K) and eukaryotic translation initiation factor 4E (eIF4E)-binding protein (4E-BP) (Hay and Sonenberg, 2004) in ON regeneration (Yang et al., 2014). Phosphorylation of 4E-BP by mTORC1 releases its binding and inhibition from eIF4E, thus to initiate cap-dependent translation. Through different mechanisms, S6K also promotes protein and lipid synthesis (Hannan et al., 2003; Duvel et al., 2010). Although they are both involved in protein synthesis, previous studies suggested that S6K regulates cell size but not cell division (Ohanna et al., 2005), whereas 4E-BP controls cell proliferation but not cell size (Dowling et al., 2010). In RGC neurons, we found that over-expression of the constitutively active mutant of S6K1 significantly increases RGC cell size after ON crush but only promote axon regeneration to a small degree (Yang et al., 2014). Together with the similar effect of TSC deletion, which activates mTORC1 to a greater extent than PTEN deletion but results in very little axon regeneration (Park et al., 2008), we conclude that mTORC1 activation itself is necessary for axon regeneration but has only minimal effect on initiating axon regeneration. Another evidence to support the necessary role of mTORC1 in axon regeneration comes from 4E-BP1-4A mutant, which cannot be inhibited by mTORC1 since it cannot be phosphorylated, as it largely blocks PTEN KO-induced axon regeneration (Yang et al., 2014). However, double deletion of 4E-BP1 and 4E-BP2 in RGCs does not promote axon regeneration, indicating the necessary but insufficient role of 4E-BP inhibition by mTORC1 in axon regeneration. The possibility that other substrates of mTORC1 in addition to S6K1 and 4E-BP may contribute to axon regeneration cannot be rule out, however, we do favor the idea that the mTORC1 pathway essentially plays a necessary role in axon regeneration which requires a key permissive signal from unidentified effectors downstream of PTEN to trigger the neuron-intrinsic growth machinery.

## AKT1 and AKT3 Are the Predominant Isoforms of AKT in RGCs and Display Different Effects on On Regeneration

AKT is downstream of PTEN/PI3K but upstream of mTORC1. AKT1 and AKT2 are widely expressed in almost any tissues, however, AKT3 is the predominant isoform in brain (Easton et al., 2005) and retina (Miao et al., 2016). Consistently, AKT1 deletion reduces whole body size and AKT2 deletion results in diabetes-like syndrome (Cho et al., 2001a,b). Deletion only of AKT3 reduces brain size (Easton et al., 2005), indicating a specific role of AKT3 in CNS growth control. We determined quantitatively the expression levels of the three AKT isoforms in RGCs and their distinct roles in axon regeneration, which provides strong evidence of the unique properties of AKT3 in retina: AKT1 and AKT3 are the major isoforms of AKTs in RGCs and activation of AKT3 promotes significantly greater RGC survival and ON regeneration than AKT1, presumably through its unique ability to activate mTORC1 (higher pS6) in retina (Miao et al., 2016). This is consistent with the results in brain, as AKT3 but not AKT1 deletion, decreases pS6 significantly (Easton et al., 2005). AKT3 may also selectively activate neuronal-specific signaling molecules that are currently unidentified.

## AKT Coordinates Positive Signals From PI3K-PDK1 and Negative Signals From mTORC2 in Regulating mTORC1 Activation and GSK3β Phosphorylation for Axon Regeneration

In addition to T308 phosphorylation by PI3K-PDK1, AKT is also phosphorylated at S473 by mTORC2 (Hresko and Mueckler, 2005; Sarbassov et al., 2005; Guertin et al., 2006). mTORC2 is activated by PI3K in an uncharacterized way but depends on ribosome (Zinzalla et al., 2011) and its activation promotes cell survival and actin cytoskeleton dynamics (Jacinto et al., 2004). Like mTORC1, mTORC2 also plays a role in lipogenesis (Lamming and Sabatini, 2013; Yao et al., 2013). It is not clear how mTORC1 and mTORC2 interact to determine multiple downstream cellular events. AKT-pS473 enhances AKT-T308 phosphorylation (Scheid et al., 2002; Yang et al., 2002) and blocking S473 phosphorylation decreases AKT-T308 phosphorylation (Hresko and Mueckler, 2005; Sarbassov et al., 2005; Guertin et al., 2009; Yuan et al., 2012; Carson et al., 2013).

We confirmed the phosphorylation of AKT-T308 and the kinase activity of AKT are essential for axon regeneration by overexpression kinase dead mutant or T308A mutant of AKT in RGCs; whereas AKT-S473A mutant results in even more axon regeneration than wild type AKT, indicating the negative role of AKT-S473 phosphorylation in axon regeneration (Miao et al., 2016). This surprising finding implies that pT308 and pS473 of AKT may have different substrates or regulate the same substrates differentially, to allow their opposite roles in axon regeneration. Interestingly, pAKT-S473 regulates β-cell proliferation whereas pAKT-T308 controls β-cell (Hashimoto et al., 2006; Gu et al., 2011), possibly through different downstream effectors (Jacinto et al., 2004, 2006; Guertin et al., 2006; Yang et al., 2006; Gu et al., 2011). Phosphorylation of glycogen synthase kinase 3β-S9 (GSK3β-S9) by AKT inhibits GSK3β activity, which is critical for neuronal polarization, axon branching and axon growth (Kim Y.T. et al., 2011). The significantly increased pGSK3β-S9 after blocking mTORC2 or overexpression of AKT3-S472A mutant suggests that GSK3β is one of the AKT effectors that are differentially regulated by pAKT-T308 and pAKT-S473 (Miao et al., 2016). The results of our studies using GSK3β-S9A mutant and GSK3β KO mice further proved the inhibitory role of GSK3β in axon regeneration (Miao et al., 2016), thus to definitively resolve the contradictory results in the literature regarding the role of GSK3β in CNS axon regeneration (Dill et al., 2008; Abe et al., 2010; Christie et al., 2010; Saijilafu Hur et al., 2013; Gobrecht et al., 2014). More interestingly, Guo et al identified eIF2B𝜀 as an important downstream effector of GSK3β for axon regeneration (Guo et al., 2016), indicating the critical role of translation regulatory machinery and protein synthesis in axon regeneration.

mTORC2 and pAKT-S473 are necessary for PTEN deletion-induced tissue overgrowth in prostate cancer of mice (Guertin et al., 2009) and eyes of drosophila (Hietakangas and Cohen, 2007). Thus blocking mTORC2 and pAKT-S473 in PTEN KO mice may allow us to minimize their deleterious tumorigenic effect but boost PTEN/AKT’s regeneration-promoting effect. mTORC1 inhibition (deletion of *RPTOR* or *mTOR*, over-expression of dominant negative mutant S6K1-DN or 4E-BP1-4A) decreased AKT3-induced ON regeneration (Miao et al., 2016), consistent with our conclusion that mTORC1 is necessary for AKT-induced axon regeneration. In summary, mTORC1 activation and GSK3β inhibition act in parallel and synergistically downstream of AKT to promote CNS axon regeneration as we and others have shown (Guo et al., 2016; Miao et al., 2016).

## Feedback Inhibition of PI3K-AKT Mediated by mTORC1-S6K1 and AKT-Independent Pathways

Proper translational control is crucial for normal cell growth. The increased protein synthesis induced by PI3K-mTORC1 activation needs to be balanced by an antagonistic mechanism. It has previously been shown that a negative feedback loop involved with S6K1 and insulin receptor substrate 1 (IRS-1) reduces activities of PI3K and its downstream effectors (Laplante and Sabatini, 2012). Indeed we detected decreased AKT phosphorylation and axon regeneration in PTEN KO mice after overexpression of S6K1 (Yang et al., 2014). Possibly through a similar mechanism, S6K inhibits axon regeneration in *C. elegans* (Hubert et al., 2014) and inhibition of S6K1 promotes corticospinal tract regeneration in mice (Al-Ali et al., 2017). It would not be surprising if additional balancing mechanisms can fine-tune the growth control loop of PI3K-AKT-mTORC1-PI3K. This feedback inhibition keeps AKT activation at minimum even after PTEN deletion (Laplante and Sabatini, 2012; Yang et al., 2014), suggesting AKT-independent signals downstream of PTEN for axon regeneration. PTEN deletion-induced PIP3-dependent signaling includes many AKT-independent pathways (Lien et al., 2017). In addition, PTEN can dephosphorylate focal adhesion kinase (FAK) and Shc, and deletion of PTEN activates FAK, RAS, and ERK (Godena and Ning, 2017). Furthermore, PTEN is also present in the nucleus to play a non-catalytic role in chromosomal instability and DNA repair (Shen et al., 2007; Song et al., 2011). Elucidation of these PTEN-dependent but AKT-independent pathways in axon regeneration will be an important future direction for the field.

## Neuronal Survival and Axon Regeneration

Neuronal survival is an obvious prerequisite for axon regeneration. But our observation is that the increased neuron survival is not invariably linked with proportionately greater axon regeneration and this is consistent with others findings (Benowitz et al., 2015). For example, ON crush injured RGC survival can be increased significantly by inhibition of apoptosis, deleting tumor suppressor genes or by manipulating ER stress, but these manipulations do not induce more ON regeneration (Goldberg et al., 2002a; Park et al., 2008; Hu et al., 2012). On the other hand, spinal cord injury does not cause significant death of corticospinal neurons (Nielson et al., 2010, 2011), but they fail to regenerate axons (Schwab and Bartholdi, 1996; Goldberg et al., 2002b; Fitch and Silver, 2008). These results indicate that the neuronal intrinsic growth signals for axon regeneration is different to the signals for neuron survival. But no convincing evidence proves a direct causative relationship between these two events cannot totally exclude the possibility that more RGC survival contributes to more potent axon regeneration. PTEN deletion or AKT activation and their signaling effectors are normally related to both intrinsic growth control and cell survival, suggesting partially overlapping functionalities of these two events. Currently we can only allow a small percentage of surviving RGCs to regenerate their axons and different subtypes of RGCs have different regeneration abilities (Duan et al., 2015). Elucidating the mechanisms caused this difference will be a hot topic in the field to maximize RGC axon regeneration.

## Axonal mRNA Translation and Axon Regeneration

The importance of localized protein synthesis in peripheral and central axon regeneration has been demonstrated *in vitro* and *in vivo* (Willis and Twiss, 2006; Jung et al., 2012; Baleriola et al., 2014; Perry and Fainzilber, 2014). And certain components of translation machinery including pS6 and 4E-BP1 have been detected in rat regenerating spinal cord (Kalinski et al., 2015). We also found that wildtype AKT and AKT-S473A mutant were localized in RGC axons whereas AKT mutants that cannot promote axon regeneration were excluded from RGC axons (Miao et al., 2016). Is the axonal AKT related with axonal mRNA translation? If so, does the regeneration phenotype caused by AKT activation rely on local protein synthesis in axons? These are very intriguing questions for future studies to investigate the significance of axonal protein synthesis and axonal signal transduction in axon regeneration.

## Conclusion and Future Perspective

In summary, genetic manipulations specifically in RGCs provide a molecular dissection of the PTEN, PI3K-AKT-mTORC1/GSK3β, and PI3K-mTORC2-AKT-mTORC1/GSK3β pathways and definitively determine the linear and parallel signals that contribute to CNS axon regeneration (**Figure 2**). The balance between mTORC1 and mTORC2’s activities after PT3K activation converges on AKT phosphorylation of T308 and S473, which in turn control the activation of mTORC1 and inhibition of GSK3β that act in parallel and synergistically downstream of AKT to promote potent CNS axon regeneration. mTORC1/S6K also functions as feedback inhibition of PI3K signaling (Laplante and Sabatini, 2012) to keep AKT and mTORC1 on check (Yang et al., 2014), which suggests that another proactive signal originating from PTEN deletion may trigger the neuron-intrinsic growth capability (Hu, 2015). It is extremely intriguing scientifically and critical clinically to identify these permissive signals of axon regeneration and elucidate the mechanisms by which they are cross regulated with the necessary mTORC1 signals. The increased understanding of the complicated cross-regulation and feedback-control mechanisms involved in PTNE-PI3K-AKT-mTORC1/2 will certainly inspire more studies on these critical growth control mechanisms, which will eventually lead to safe and effective therapeutic strategies for CNS injury, and to isolate them from targets that mediate deleterious effects.

## Author Contributions

YH and JZ contributed to bibliographical search, writing, and figure design. HH, DY, and YS wrote the paper.

## Conflict of Interest Statement

The authors declare that the research was conducted in the absence of any commercial or financial relationships that could be construed as a potential conflict of interest.
